# Association between Laughter and Lifestyle Diseases after the Great East Japan Earthquake: The Fukushima Health Management Survey

**DOI:** 10.3390/ijerph182312699

**Published:** 2021-12-02

**Authors:** Eri Eguchi, Tetsuya Ohira, Hironori Nakano, Fumikazu Hayashi, Kanako Okazaki, Mayumi Harigane, Narumi Funakubo, Atsushi Takahashi, Kanae Takase, Masaharu Maeda, Seiji Yasumura, Hirooki Yabe, Kenji Kamiya, on behalf of the Fukushima Health Management Survey Group

**Affiliations:** 1 Department of Epidemiology, Fukushima Medical University School of Medicine, Fukushima 960-1295, Japan; teoohira@fmu.ac.jp (T.O.); h-nakano@fmu.ac.jp (H.N.); fhayashi@fmu.ac.jp (F.H.); naru-23@fmu.ac.jp (N.F.); 2 Radiation Medical Science Center for the Fukushima Health Management Survey, Fukushima Medical University, Fukushima 960-1295, Japan; kana-oka@fmu.ac.jp (K.O.); harigane@fmu.ac.jp (M.H.); junior@fmu.ac.jp (A.T.); takase@fmu.ac.jp (K.T.); masagen@fmu.ac.jp (M.M.); yasumura@fmu.ac.jp (S.Y.); hyabe@fmu.ac.jp (H.Y.); kkamiya@fmu.ac.jp (K.K.); 3 Department of Physical Therapy, Fukushima Medical University School of Health Sciences, Fukushima 960-8516, Japan; 4 Department of Public Health, Fukushima Medical University School of Medicine, Fukushima 960-1295, Japan; 5 Department of Gastroenterology, Fukushima Medical University School of Medicine, Fukushima 960-1295, Japan; 6 Department of Community Health and Public Health Nursing, Fukushima Medical University School of Nursing, Fukushima 960-1295, Japan; 7 Department of Disaster Psychiatry, Fukushima Medical University School of Medicine, Fukushima 960-1295, Japan; 8 Department of Neuropsychiatry, Fukushima Medical University School of Medicine, Fukushima 960-1295, Japan; 9 Research Institute for Radiation Biology and Medicine, Hiroshima University, Hiroshima 734-8553, Japan

**Keywords:** laughter, disaster, Great East Japan Earthquake, lifestyle diseases, cardiovascular diseases, evacuation

## Abstract

We investigated the association between the frequency of laughter and lifestyle diseases after the Great East Japan Earthquake. We included 41,432 participants aged 30–89 years in the Fukushima Health Management Survey in fiscal year 2012 and 2013. Gender-specific, age-adjusted and multivariable odds ratios of lifestyle diseases were calculated using logistic regressions stratified by evacuation status. Those who laugh every day had significantly lower multivariable odds ratios for hypertension (HT), diabetes mellitus (DM) and heart disease (HD) for men, and HT and dyslipidemia (DL) for women compared to those who do not, especially in male evacuees. The multivariable odds ratios (95% confidence intervals) of HT, DM and HD (non-evacuees vs. evacuees) for men were 1.00 (0.89–1.11) vs. 0.85 (0.74–0.96), 0.90 (0.77–1.05) vs. 0.77 (0.64–0.91) and 0.92 (0.76–1.11) vs. 0.79 (0.63–0.99), and HT and DL for women were 0.90 (0.81–1.00) vs. 0.88 (0.78–0.99) and 0.80 (0.70–0.92) vs. 0.72 (0.62–0.83), respectively. The daily frequency of laughter was associated with a lower prevalence of lifestyle disease, especially in evacuees.

## 1. Introduction

Scholars have recently reported the health benefits of daily laughter. Previous studies have shown that laughter is associated with a lower prevalence of cardiovascular disease [1] and may decrease the risk of such diseases [2]. Experimental studies have also demonstrated that laugher moderates stress, improves the immune system [3,4], decreases allergic responses [5], reduces the increase in postprandial blood glucose in patients with diabetes mellitus [6] and improves blood vessel function [7]. Moreover, an increasing number of interventional studies have revealed positive effects of laughter on depression, insomnia, self-rated health and hemoglobin A1c levels [8,9,10].

The Great East Japan Earthquake and the subsequent Fukushima Daiichi nuclear disaster in March 2011 constituted one of the most destructive catastrophes in Japan to date. Due to radiation concerns, most residents in nearby towns had to evacuate and, consequently, suffered long-lasting anxiety. Shortly after the disaster, the Fukushima Health Management Survey was launched [11,12]. Similar to previous studies [13,14,15,16,17,18,19,20,21,22,23,24,25], the survey’s results showed that after the disaster, the prevalence of people with cardiovascular risk factors [26]—including overweight [27], hypertension [28], diabetes mellitus [29], dyslipidemia [30] and metabolic syndrome [31]—increased among community residents. At the same time, as in previous studies [32] or even more, community residents exhibited psychological distress [33,34,35], particularly among evacuees [26,36].

The favorable effects of laughter on health may be associated with better health even after this disaster. Recently, the positive effects of laughter on human health have been recognized [37,38], and scholars have suggested that the lack of positive factors may be a more critical determinant to develop lifestyle diseases than the presence of negative factors. For example, previous studies have shown that depression on mortality and functional decline might result from the absence of positive factors rather than the presence of negative ones [39,40]. To our knowledge, the effects of laughter on lifestyle diseases after a disaster have not been examined. There may be a strong association between laughter and lifestyle-related diseases under high-stress conditions, such as evacuation due to a disaster. Therefore, in this study, we investigated the association between the frequency of laughter as a positive psychological factor and lifestyle diseases after the Great East Japan Earthquake based on the hypothesis that the prevalence of lifestyle-related diseases is lower in the group who laugh every day and that the association may be particularly strong among those who experienced evacuation.

## 2. Materials and Methods

### 2.1. Study Population

**Figure 1:**
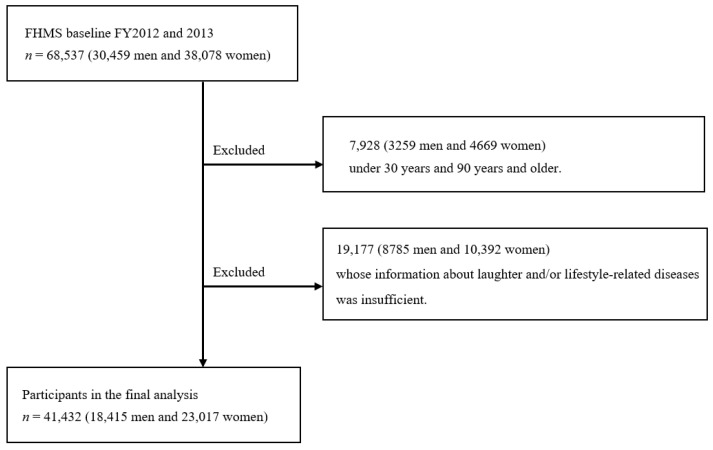
Flow chart of participant selection for the present study. Association between laughter and lifestyle diseases after the Great East Japan Earthquake: The Fukushima Health Management Survey.

The Mental Health and Lifestyle Survey was a part of the Fukushima Health Management Survey started after the Great East Japan Earthquake in 2011. The Fukushima Health Management Survey is a large cohort study that monitors the long-term health of residents in Fukushima Prefecture after the earthquake and promotes their future well-being. The details of this survey are described elsewhere [11,12]. The subjects of the survey were officially registered as residents of the evacuation zone designated by the government (including those who evacuated or moved to other prefectures) between 11 March 2011 and 1 April 2012, regardless of their age. The areas included Hirono Town, Naraha Town, Tomioka Town, Kawauchi Village, Okuma Town, Futaba Town, Namie Town, Katsurao Village, Minamisoma City, Tamura City, Kawamata Town, Iitate Village and a part of Date City. We targeted all residents of the evacuation areas in the survey. Questionnaires were mailed to residents in January 2012, 2013 and 2014, and the deadline for responses was set at six months. Out of the 68,537 respondents of the data of fiscal year 2012 and 2013, we excluded 7928 people under the age of 30 and over the age of 89. We also excluded 19,117 people who had a lack of information on the frequency of laughter and/or the diagnosis of lifestyle-related diseases. Ultimately, 41,432 respondents were included in the analysis (Figure 1). The survey participants were informed in writing; the results were totaled and reported after analysis, and only those who returned the self-recorded questionnaire were considered to have provided consent to participate in the study. This study was approved by the Ethics Committee of Fukushima Medical University (#1316, #2148 and IPPAN 2020-239).

### 2.2. Measurements

#### 2.2.1. Frequency of Laughter

We assessed the daily frequency of laughter by a single question, “How often do you laugh out loud?”. Possible responses included “almost every day”, “1–5 days per week”, “1–3 days per month”, and “almost never”. This variable was dichotomized. Those who responded “almost every day” were categorized as people who laugh every day, and those who responded otherwise were categorized as people who do not laugh every day. This measurement has been used in previous studies in Japan [1,41], and reliability was also assessed in this study.

#### 2.2.2. Lifestyle Diseases

We assessed the lifestyle diseases of cancer, stroke, heart disease, hypertension, diabetes mellitus and dyslipidemia via questionnaires. A single question was, “Have you ever been diagnosed with [a disease]?” The respondents answered either “Yes” or “No”.

#### 2.2.3. Experience of Evacuation

We asked the question, “How have you changed your living condition as a result of the earthquake?” to check the experience of evacuation of participants. Possible answers were “evacuation shelter”, “temporary housing”, “rental house/apartment”, “relative’s house”, “owned house”, or “other”. Those who answered, “evacuation shelter” and “temporary housing” were defined as people who had experienced evacuation. For those without this information, we asked “Where do you currently live?”. Possible answers were “municipally subsidized rental housing”, “temporary housing”, “restoration public housing”, “rented house/apartment”, “relative’s house”, “owned house”, and “other”. Those who answered, “Municipally subsidized rental housing”, “Temporary housing”, “Restoration public housing”, were defined as people who had experienced evacuation; that is, they changed their living environment that would affect their lives more after the disaster.

#### 2.2.4. Lifestyle Factors

We obtained data on height and weight by the questionnaire, and body mass index (BMI) was calculated as weight (kg)/height (m^2^). Smoking status was obtained and categorized into current and non-smokers, including past smokers. The alcohol consumption was determined and categorized into current drinkers (more than once a month) and non-drinkers, including past drinkers. Physical activity status was obtained by a single question, “Do you usually do exercise?” Possible answers included “almost every day”, “2–4 times per week”, “once a week”, and “almost never”. The respondents’ quality of sleep was assessed by asking a single question, “Are you satisfied with the quality of your sleep over the past month (regardless of sleep duration)?”. On the questionnaire, possible answers were “Satisfied”, “Slightly dissatisfied”, “Quite dissatisfied”, and “Very dissatisfied or haven’t slept at all”.

#### 2.2.5. Other Variables

The patient’s subjective health was assessed by a single question, “How is your current health condition?” and possible answers were “very good”, “good”, “normal”, “bad”, and “very bad”. Those who answered, “very good”, “good”, and “normal”, were categorized as having a better subjective health. Psychological distress was assessed using the Japanese version of the Kessler 6 (K6) scale [42,43], and those with a score of ≥ 13 were defined as having psychological distress. Cronbach’s alpha for K6 was 0.92 [43,44,45]. Trauma reactions were also assessed by the Japanese version of the Posttraumatic Stress Disorder (PTSD) Checklist–Stressor-Specific Version (PCL-S) [46,47,48]. A total score of ≥ 44 was classified as a sign of probable PTSD. Cronbach’s alpha for PCL-S was 0.96 [46,49]. Job status was assessed by asking a single question, “Please tell us about your current work style” and possible answers were “full-time”, “part-time”, and “unemployed”. Connection with others was assessed by “How many (1. relatives or siblings or 2. friends) do you have that you can talk to about personal matters without hesitation?” and “How many (1. relatives or siblings or 2. friends) do you feel close to that you can ask for help?” and possible answers were “0”, “1”, “2”, “3–4”, “5–8”, and “more than 9”.

### 2.3. Statistical Analysis

The gender-specific characteristics of the respondents were described according to the categories of the frequency of laughter. Multiple analyses were conducted for those who laugh 1–5 days per week, 1–3 days per month, almost never compared to those who laugh every day according to each factor. We derived the *p*-values for the trend analysis from a regression model for continuous variables, and a logistic model for dichotomized variables according to the categories of laughter adjusted by age. Using logistic regressions, we calculated gender-specific, age-adjusted odds ratios, multivariable odds ratios, and the 95% confidence interval of hypertension, diabetes mellitus, dyslipidemia, cancer, stroke, or heart disease for those who laugh almost every day compared to those who do not. The same analyses were performed and stratified by the presence or absence of an evacuation experience. The covariates included age (continuous), body mass index (continuous), smoking status (non-smoker, current smoker, or missing), drinking status (current drinker, non-drinker, or missing), habitual physical activity (almost every day, 2–4 times per week, about once a week, almost never, or missing), quality of sleep (satisfied, somewhat unsatisfied, highly unsatisfied, very unsatisfied or could not sleep, or missing), mental health distress (yes, no, or missing), job status (full-time, part-time, unemployed, or missing), connection with others (one or more closer friend or relatives, or missing) for the analysis of hypertension, diabetes mellitus and dyslipidemia, and history of hypertension, diabetes mellitus and dyslipidemia (yes, no, or missing) in addition to other variables for the analysis of stroke, heart disease or cancer. We used the SAS software version 9.4 (SAS Institute, Cary, North Carolina, USA) for all statistical analyses. All probability values for statistical tests were two-tailed and *p* < 0.05 was regarded as statistically significant.

## 3. Results

### 3.1. Characteristics of Participants

Among the 41,432 individuals, 6383 (34.6%) men and 6286 (27.3%) women reported hypertension, 2410 (13.1%) men and 1567 (6.8%) women for diabetes mellitus, 2604 (13.1%) men and 3169 (13.8%) women for dyslipidemia, 776 (4.2%) men and 514 (2.2%) women for cancer, 370 (2.0%) men and 213 (0.9%) women for stroke, and 1597 (8.7%) men and 1349 (5.9%) women for heart disease. Table 1 shows the characteristics of participants according to the frequency of daily laughter. The proportion of those who laugh almost every day was 23.1% in men and 28.6% in women. The proportion of those who experienced evacuation was 47.1% in men and 48.2% in women. Compared to those who laugh every day, those who do not laugh every day were older, had lower BMI, had lower prevalence of habitual alcohol drinker and physical activity, better subjective health, full-time job, had higher prevalence of smoker, unsatisfied sleep quality, mental health distress, traumatic symptom, and experience of evacuation in men; and were older, had lower prevalence of habitual alcohol drinker and physical activity, better subjective health, full-time job, and higher prevalence of smoker, unsatisfied sleep quality, mental health distress, traumatic symptom, and experience of evacuation in women (*p* for trend < 0.01).

### 3.2. Associations between Frequency of Laughter and Lifestyle Diseases

Table 2 shows the gender-specific association between the frequency of laughter and lifestyle diseases. For men, compared to those who do not laugh every day, the participants who laugh every day had lower age and gender-adjusted odds ratios for hypertension, diabetes mellitus, dyslipidemia and heart disease. Those associations remained statistically significant even after adjusting for other related variables of lifestyle and diseases like diabetes mellitus and heart disease. The multivariable odds ratios were 0.84 (0.75–0.94) for diabetes mellitus and 0.86 (0.75–1.00) for heart disease. In women, the participants who laugh every day had lower age and gender-adjusted odds ratios for hypertension, diabetes mellitus, dyslipidemia, stroke and heart disease compared to those who do not. This association remained statistically significant even after adjusting for other variables related to lifestyle, diseases, hypertension and dyslipidemia. The multivariable odds ratios were 0.89 (0.83–0.97) for diabetes mellitus and 0.76 (0.69–0.84) for dyslipidemia. On multivariate analysis, men who laugh every day had higher odds ratio for cancer compared to those who did not.

### 3.3. Associations between Frequency of Laughter and Lifestyle Diseases According to Evacuation Experiences

Table 3 shows the association between the frequency of laughter and lifestyle diseases stratified by evacuation experiences. The magnitude of associations was slightly larger for evacuees with hypertension, diabetes mellitus and dyslipidemia compared to non-evacuees, particularly men. Moreover, an association was evident and stronger for men with heart disease with evacuation experiences compared those without. For stroke, the association was not significant in either men or women for multivariable analysis. The multivariable odds ratios for hypertension, diabetes mellitus and dyslipidemia for men (non-evacuees vs. evacuees) were 1.00 (0.89–1.11) vs. 0.85 (0.74–0.96), 0.90 (0.77–1.05) vs. 0.77 (0.64–0.91) and 0.95 (0.81–1.10) vs. 0.92 (0.78–1.07), respectively; for women, they were 0.90 (0.81–1.00) vs. 0.88 (0.78–0.99), 0.84 (0.70–1.01) vs. 0.93 (0.77–1.13) and 0.80 (0.70–0.92) vs. 0.72 (0.63–0.83), respectively. The multivariable odds ratios for heart disease among men (non-evacuees vs. evacuees) was 0.92 (0.76–1.11) vs. 0.79 (0.63–0.99).

## 4. Discussion

Those who experienced evacuation had a lower prevalence of those who laugh daily. Compared to those who do not laugh every day, those who laugh every day had a lower prevalence of hypertension, diabetes mellitus, dyslipidemia and heart disease after the Great East Japan Earthquake, and the magnitude of associations was larger for evacuees especially men.

To the best of our knowledge, this is the first study to examine the relationship between the frequency of laughter and lifestyle-related diseases stratified by evacuation experience after a large-scale disaster. So far, several studies have reported the deterioration of health status after major disasters, both physically and psychologically [13,14,15,16,17,18,19,20,21,22,23,24,25]. Similarly, in addition to the mental health deterioration [49], after the Great East Japan Earthquake, increases in body weight [27], hypertension [28], diabetes mellitus [29], dyslipidemia [30] and metabolic syndrome were reported [31]. Furthermore, there are few reports on the association between the daily frequency of laughter and lifestyle diseases such as hypertension, diabetes mellitus and dyslipidemia. For example, Hayashi et al. reported that the daily frequency of laughter was associated with a lower prevalence of cardiovascular diseases among a population of about 20,000 men and women aged 65 years and above [1]. In addition, Sakurada et al. investigated the association between the daily frequency of laughter and the all-cause mortality and cardiovascular disease [2]. In the current study, we proved our hypothesis by showing that those who laugh every day had a lower prevalence of lifestyle-related disease that increase after a major disaster. In addition, an important point of our results is that the association might be stronger in those who experienced evacuation, particularly among men. There are many possible factors for this. After a large-scale disaster, victims’ health is threatened by a variety of issues, such as lifestyle changes, changes from loss of social community due to evacuation, loss of close relatives, disaster trauma and stress caused by these factors, which may lead to the development of lifestyle diseases. In fact, in the present study, it is shown that people who laugh less frequently were more likely to have experienced evacuation. However, laughter has been reported to have stress-reducing effects increasing physical activity, mental health benefits and improvement in other related lifestyle and social interaction [50]. These effects are considered to have enabled evacuees to prevent lifestyle-related diseases through the revival of a diminished social community, the reduction of trauma and stress, and other effects on improving lifestyle habits and mental health status. The results of this study confirm that the association between laughter and lifestyle-related diseases is weakened when adjusted for social factors. This indicates that the social connections that are generated by laughter, or, conversely, laughter that is thought to increase in frequency due to social connections, may have a close relationship with the prevention of lifestyle-related diseases. Previous studies have reported a higher risk of developing lifestyle diseases and cardiovascular disease after a major disaster in evacuees than in non-evacuees [27,28,29,30,31,36]. The value of laughter after a major disaster increases the stronger association among evacuees between laughter and lifestyle and cardiovascular disease. In the current study, we found no significant association for stroke. The results may be influenced by the lower number of people who had a stroke during the study period than that of other diseases. It is possible that the number of cases of stroke may increase after more time has passed since the disaster and an association may be found. The frequency of daily laughter has been reported to increase with social support, social networks, subjective well-being called “ikigai”, conversations with others and healthy diets including fish, beans, household items, fruits and vegetables [9,41,50,51,52]. In addition, it is becoming clear that laughter programs, such as laughter yoga and other laughter activities, also increase those favorable lifestyle choices [9]. Laughing on a daily basis and being in an environment where one can laugh during challenging situations is important for living without lifestyle diseases. In the present study, we found that men who laugh everyday were more likely to have cancer than those who do not. However, since laughing is unlikely to be a cause of cancer, we inference that many of the men with cancer may have become aware of the benefits of laughter after receiving treatment and began to incorporate laughter into their daily lives. This point needs to be explored in more detail in a longitudinal study.

We faced the following limitations of this study. First, as we conducted a cross-sectional study, the causal relationship is unclear. Therefore, we are preparing a prospective study for the next analysis. Second, in our study, lifestyle diseases and cardiovascular diseases were confirmed by self-administrated questionnaires, not measurements during health checkups, which could lead to non-differential measurement errors and attenuation of the associations. Third, the overall response rate to the questionnaires was not very high (40.7%), and there were some differences in the distribution of ages between the study sample and the target population. Therefore, the results of the study may not reflect all of the evacuation areas specified by the government.

## 5. Conclusions

After the Great East Japan Earthquake, daily frequency of laughter was associated with a lower prevalence of hypertension, diabetes mellitus, dyslipidemia and heart disease. The association was particularly strong among men who had experienced evacuation, suggesting that positive factors such as laughter may be effective in the prevention of lifestyle diseases under stressful conditions, such as after a major disaster. Further longitudinal or intervention studies are needed to evaluate the relevant causal relationships.

## Figures and Tables

**Table 1:** Gender-specific means and proportions of characteristics for participants according to the frequency of laughter.

	Frequency of Laughter				
	Almost Every Day	1–5 Days per Week	1–3 Days per Month	Almost Never	*p* for Trend
Men					
Number, %	4258 (23.1)	7208 (39.1)	3938 (21.4)	3011 (16.4)	
Age, years old	61.9 ± 15.4	61.6 ± 14.6	62.4 ± 13.3	62.3 ± 13.3	<0.0001
30–40 years old, %	13.2	11.5 ^†^	7.5 ^†^	7.1 ^†^	<0.0001
40–50 years old, %	10.6	11.2	10.1	10.0	0.17
50–60 years old, %	12.8	15.1 ^†^	18.3 ^†^	19.8 ^†^	<0.0001
60–70 years old, %	24.6	28.5 ^†^	32.8 ^†^	33.2 ^†^	<0.0001
70–80 years old, %	27.6	23.9	21.5	19.7	<0.0001
80 years old and older, %	11.2	9.8	9.7	10.3	0.20
Body mass index, kg/m^2^	24.4 ± 3.9	24.2 ^†^ ± 3.5	24.2 ^†^ ± 3.8	23.9 ^†^ ± 3.7	<0.0001
Smoker, %	28.0	26.7	30.0	29.2	0.003
Habitual alcohol drinker, %	65.7	68.4 ^†^	67.1	58.6 ^†^	<0.0001
Habitual physical activity, %	50.9	43.4 ^†^	34.9 ^†^	25.7 ^†^	<0.0001
Unsatisfied with sleep quality, %	7.8	11.8 ^†^	17.9 ^†^	29.7 ^†^	<0.0001
Better subjective health, %	89.9	84.0 ^†^	77.8 ^†^	63.6 ^†^	<0.0001
Mental health distress, %	3.8	6.7 ^†^	11.5 ^†^	25.3 ^†^	<0.0001
Traumatic symptom, %	10.5	13.2 ^†^	17.5 ^†^	32.2 ^†^	<0.0001
Full-time job, %	51.3	47.4 ^†^	44.5 ^†^	38.4 ^†^	<0.0001
Experience of evacuation	42.2	45.8 ^†^	50.2 ^†^	53.3 ^†^	<0.0001
Women					
Number, %	6585 (28.6)	9702 (42.2)	4338 (18.9)	2392 (10.4)	
Age, years old	59.6 ± 13.3	61.1 ^†^ ± 15.3	62.1 ^†^ ± 14.3	63.6 ^†^ ± 14.3	<0.0001
30–40 years old, %	16.9	13.2 ^†^	10.0 ^†^	7.7 ^†^	<0.0001
40–50 years old, %	13.3	11.4 ^†^	9.8 ^†^	9.1 ^†^	<0.0001
50–60 years old, %	14.4	15.5	17.6 ^†^	19.3 ^†^	0.0001
60–70 years old, %	21.3	26.6 ^†^	30.3 ^†^	28.3 ^†^	<0.0001
70–80 years old, %	23.7	21.9	20.5	19.5	<0.0001
80 years old and older, %	10.4	11.5	11.8	16.1 ^†^	<0.0001
Body mass index, kg/m^2^	23.3 ± 3.9	23.3 ± 4.1	23.4 ± 4.3	23.1 ^†^ ± 4.1	0.12
Smoker, %	7.2	7.1	9.1 ^†^	10.0 ^†^	<0.0001
Habitual alcohol drinker, %	26.4	27.1	25.8	23.2 ^†^	0.008
Habitual physical activity, %	43.9	39.4 ^†^	28.8 ^†^	21.7 ^†^	<0.0001
Unsatisfied with sleep quality, %	9.8	15.7 ^†^	25.5 ^†^	33.7 ^†^	<0.0001
Better subjective health, %	88.4	81.4 ^†^	70.9 ^†^	57.8 ^†^	<0.0001
Mental health distress, %	4.8	10.2 ^†^	20.5 ^†^	36.2 ^†^	<0.0001
Traumatic symptom, %	12.1	17.5 ^†^	28.0 ^†^	39.5 ^†^	<0.0001
Full-time job, %	22.8	19.2 ^†^	16.8 ^†^	15.0 ^†^	<0.0001
Experience of evacuation	41.2	48.9 ^†^	54.0 ^†^	54.2 ^†^	<0.0001

^†^ <0.05 Those who laugh almost every day as a reference category.

**Table 2:** Gender-specific age-adjusted and multivariable odds ratios of lifestyle diseases for those who laugh every day compared to those who do not laugh every day.

		Men	Women
Number		18,415	23,017
Hypertension		6383	6286
	Age-adjusted odds ratio	0.89 (0.82–0.96)	0.86 (0.80–0.92)
	Multivariable odds ratio *^a^	0.92 (0.85–1.00)	0.89 (0.82–0.96)
	Multivariable odds ratio *^c^	0.93 (0.85–1.01)	0.89 (0.83–0.97)
Diabetes mellitus		2410	1567
	Age-adjusted odds ratio	0.83 (0.74–0.92)	0.86 (0.77–0.97)
	Multivariable odds ratio *^a^	0.83 (0.74–0.94)	0.87 (0.76–1.00)
	Multivariable odds ratio *^c^	0.84 (0.75–0.94)	0.87 (0.77–1.01)
Dyslipidemia		2604	3169
	Age-adjusted odds ratio	0.83 (0.75–0.92)	0.70 (0.64–0.76)
	Multivariable odds ratio *^a^	0.93 (0.83–1.03)	0.75 (0.68–0.83)
	Multivariable odds ratio *^c^	0.93 (0.84–1.04)	0.76 (0.69–0.84)
Cancer		776	514
	Age-adjusted odds ratio	1.04 (0.88–1.24)	0.94 (0.77–1.15)
	Multivariable odds ratio *^b^	1.22 (1.01–1.47)	1.16 (0.93–1.44)
	Multivariable odds ratio *^c^	1.23 (1.02–1.48)	1.15 (0.92–1.43)
Stroke		370	213
	Age-adjusted odds ratio	0.80 (0.62–1.04)	0.62 (0.44–0.88)
	Multivariable odds ratio *^b^	0.95 (0.71–1.27)	0.81 (0.54–1.21)
	Multivariable odds ratio *^c^	0.95 (0.71–1.26)	0.80 (0.54–1.20)
Heart disease		1597	1349
	Age-adjusted odds ratio	0.73 (0.64–0.84)	0.75 (0.66–0.86)
	Multivariable odds ratio *^b^	0.86 (0.75–1.00)	0.92 (0.79–1.07)
	Multivariable odds ratio *^c^	0.86 (0.75–1.00)	0.93 (0.80–1.09)

*^a^: Multivariable adjustment of age, body mass index, smoking status, drinking status, habitual physical activity, quality of sleep, mental health distress, job status and connection with others. *^b^: Multivariable adjustment of history of hypertension, diabetes mellitus and dyslipidemia in addition to *^a^. *^c^: Multivariable adjustment of evacuation in addition to *^a^ or *^b^.

**Table 3:** Gender-specific age-adjusted and multivariable odds ratios of lifestyle diseases for those who laugh every day compared to those who do not laugh every day stratified by experience of evacuation.

		Men	Women
Non-evacuees			
Number		9734	11,924
Hypertension		3236	3142
	Age-adjusted odds ratio	0.96 (0.86–1.06)	0.87 (0.79–0.95)
	Multivariable odds ratio *^a^	1.00 (0.89–1.11)	0.90 (0.81–1.00)
Diabetes mellitus		1180	746
	Age-adjusted odds ratio	0.88 (0.76–1.02)	0.78 (0.66–0.92)
	Multivariable odds ratio *^a^	0.90 (0.77–1.05)	0.84 (0.70–1.01)
Dyslipidemia		1243	1470
	Age-adjusted odds ratio	0.83 (0.72–0.96)	0.74 (0.65–0.84)
	Multivariable odds ratio *^a^	0.95 (0.81–1.10)	0.80 (0.70–0.92)
Cancer		389	273
	Age-adjusted odds ratio	1.02 (0.81–1.29)	0.95 (0.73–1.23)
	Multivariable odds ratio *^b^	1.17 (0.91–1.52)	1.14 (0.86–1.52)
Stroke		186	112
	Age-adjusted odds ratio	0.57 (0.39–0.84)	0.47 (0.28–0.77)
	Multivariable odds ratio *^b^	0.69 (0.45–1.06)	0.66 (0.37–1.17)
Heart disease		808	624
	Age-adjusted odds ratio	0.79 (0.66–0.94)	0.68 (0.56–0.82)
	Multivariable odds ratio *^b^	0.92 (0.76–1.11)	0.82 (0.66–1.02)
Evacuees			
Number		8681	11,093
Hypertension		3147	3144
	Age-adjusted odds ratio	0.83 (0.74–0.93)	0.87 (0.78–0.97)
	Multivariable odds ratio *^a^	0.85 (0.74–0.96)	0.88 (0.78–0.99)
Diabetes mellitus		1230	821
	Age-adjusted odds ratio	0.79 (0.67–0.93)	1.00 (0.85–1.19)
	Multivariable odds ratio *^a^	0.77 (0.64–0.91)	0.93 (0.77–1.13)
Dyslipidemia		1361	1699
	Age-adjusted odds ratio	0.84 (0.73–0.98)	0.68 (0.60–0.78)
	Multivariable odds ratio *^a^	0.92 (0.78–1.07)	0.72 (0.63–0.83)
Cancer		387	241
	Age-adjusted odds ratio	1.10 (0.86–1.41)	0.93 (0.68–1.26)
	Multivariable odds ratio *^b^	1.29 (0.98–1.70)	1.17 (0.83–1.65)
Stroke		184	101
	Age-adjusted odds ratio	1.15 (0.81–1.63)	0.86 (0.53–1.40)
	Multivariable odds ratio *^b^	1.33 (0.89–1.99)	1.05 (0.59–1.86)
Heart disease		789	725
	Age-adjusted odds ratio	0.68 (0.56–0.84)	0.87 (0.73–1.06)
	Multivariable odds ratio *^b^	0.79 (0.63–0.99)	1.05 (0.85–1.30)

*^a^: Multivariable adjustment of age, body mass index, smoking status, drinking status, habitual physical activity, quality of sleep, mental health distress, job status and connection with others. *^b^: Multivariable adjustment of history of hypertension, diabetes mellitus and dyslipidemia in addition to *^a^.

## Data Availability

The datasets analyzed during the present study are not publicly available because the data from the Fukushima Health Management Survey belongs to the government of Fukushima Prefecture and can only be used within the organization.
